# Sustained platelet-sparing effect of weekly low dose paclitaxel allows effective, tolerable delivery of extended dose dense weekly carboplatin in platinum resistant/refractory epithelial ovarian cancer

**DOI:** 10.1186/1471-2407-11-289

**Published:** 2011-07-11

**Authors:** Rohini Sharma, Janet Graham, Sarah Blagden, Hani Gabra

**Affiliations:** 1 Department of Experimental Medicine, Imperial College, London, UK; 2 Department of Oncology, University of Glasgow, Glasgow, UK; 3 Ovarian Cancer Action (HHMT) Research Centre, Imperial College, London, UK

**Keywords:** ovarian cancer, resistance, chemotherapy, dose-dense, carboplatin, paclitaxel, thrombocytopaenia

## Abstract

**Background:**

Platinum agents have shown demonstrable activity in the treatment of patients with platinum resistant, recurrent ovarian cancer when delivered in a "dose-dense" fashion. However, the development of thrombocytopenia limits the weekly administration of carboplatin to no greater than AUC 2. Paclitaxel has a well-described platelet sparing effect however its use to explicitly provide thromboprotection in the context of dose dense carboplatin has not been explored.

**Methods:**

We treated seven patients with platinum resistant ovarian cancer who had previously received paclitaxel or who had developed significant peripheral neuropathy precluding the use of further full dose weekly paclitaxel.

**Results:**

We were able to deliver carboplatin AUC 3 and paclitaxel 20 mg/m^2 ^with no thrombocytopenia or worsening of neuropathic side-effects, and with good activity.

**Conclusions:**

We conclude that this regimen may be feasible and active, and could be formally developed as a "platinum-focussed dose-dense scaffold" into which targeted therapies that reverse platinum resistance can be incorporated, and merits further evaluation.

## Background

Ovarian cancer is the leading cause of death from gynaecologic malignancies in the United Kingdom, and is the fourth most common cause of cancer mortality in women [1]. Despite 70-80% overall response to initial therapy, the majority of patients will experience disease relapse and will require further chemotherapy [2,3]. Several therapeutic options are available and the decision as to which therapy to commence is dependent on the time from last platinum chemotherapy to decision to treat, known as the platinum-free interval (PFI) [4,5]. The PFI is a predictor of response not only to second-line treatment with platinum-based chemotherapy but also other active agents [6]. Stemming from the original paper by Markmann and colleagues in 1991, patients who relapse within 6 months are termed platinum resistant, those who never respond platinum refractory and those who relapse after more than one year platinum sensitive [4]. The term partially sensitive has come into use more recently to describe the patients with a PFI of 6 to 12 months. The probability of response to platinum rechallenge increases with the PFI, from > 60% in patients relapsing > 12 months since last platinum therapy to below 10% in patients relapsing within 6 months [4]. There is increasing evidence, however, that by administering platinum in a 'dose-dense' manner, resistance can be overcome resulting in significant improvements in response [7].

Dose intensity is defined as the amount of drug administered per unit of time, and can be increased in a number of ways [8]. Firstly, the dose delivered per cycle can be increased (dose intensity), secondly, the dosing interval can be reduced keeping the dose per cycle and overall dose the same (dose densification), and finally both the dosing interval can be reduced and the overall dose increased (increased dose intensity, total dose, and dose density) [9]. The concept of dose manipulation, in particular the delivery of dose dense chemotherapy is not novel and has been extensively studied in a number of tumour types in particular breast cancer where significant improvements in both disease free survival and overall survival have been reported [10]. More recently, use of adjuvant dose dense weekly paclitaxel in combination with conventional platinum has demonstrated significant improvement in progression free survival and overall survival in ovarian cancer [11].

A number of different 'dose-dense' carboplatin and paclitaxel regimens have been developed for the treatment of both front line and recurrent ovarian cancer [7,12]. Common to all these studies is the development of neurotoxicity associated with weekly paclitaxel, the reported incidence of grade 3/4 neurotoxicity ranging from 2-94%. The development of neuropathy may have a negative impact on the administered dose intensity and patients' quality of life, an important consideration in this essentially palliative population. Furthermore, platinum resistant patients with existing peripheral neuropathy or other co-morbidities such as diabetes mellitus are poor candidates for dose dense approaches that utilise either cisplatin or paclitaxel because of the risk of further deterioration of their neurological symptoms.

In the context of dose dense therapy the role of taxanes is twofold, firstly to impart a cytotoxic effect and secondly to improve the feasibility of carboplatin delivery by utilising the platelet sparing effect of paclitaxel [13,14]. Clinically, it is accepted that carboplatin given at AUC > 2 weekly is non-feasible due to development of dose-limiting thrombocytopenia, whereas the addition of weekly paclitaxel, 70-90 mg/m^2^, allows higher doses of carboplatin to be delivered weekly [15-17]. However this dose dense schedule sits close to the limits of feasibility, and may reduce the ability to subsequently integrate targeted therapies, directed at reversing platinum resistance. We therefore chose to deliver a lower but sufficient dose of paclitaxel to prevent thrombocytopenia when delivering dose dense platinum in order to minimise neuropathy in those patients with co-morbidities such as diabetes or pre-existing (or developing) taxane neuropathy.

In this paper, we hypothesised that the platelet sparing effect of paclitaxel occurs at doses lower than that required for response. Therefore, patients receiving weekly dose dense therapy who developed neuropathy necessitating the reduction of paclitaxel dose were afforded the opportunity to continue chemotherapy at reduced paclitaxel dose whilst maintaining platinum dose density. We describe here a case series of patients receiving dose dense weekly carboplatin AUC 3 with paclitaxel 20 mg/m^2 ^day 1, 8, 15 q4 weekly where reductions in paclitaxel dose were required due to clinical indication. We demonstrate that the addition of low dose paclitaxel allows delivery of weekly dose dense carboplatin AUC 3 by maintaining platelet counts without compromising dose dense carboplatin mediated tumour response. We also discuss the concept of a platinum-focussed dose dense "scaffold" that may allow the more feasible integration of single or multiple targeted therapies aimed at reversing platinum resistance specifically, potentially bringing us closer to effective platinum resistance reversal in the clinic.

## Methods

Seven consecutive patients with platinum resistant epithelial ovarian cancer (relapse < 6 months from last platinum), with grade ≥ 2 neuropathic symptoms at the time of commencement of dose dense therapy or who developed neuropathy whilst receiving weekly combination carboplatin AUC 3 and paclitaxel 70 mg/m^2^, were treated with carboplatin (AUC 3) and paclitaxel (20 mg/m^2^) administered day 1, 8, 15 q4weekly. Patients were treated at the Department of Medical Oncology, Hammersmith Hospital, London, between 2009 and 2010.

Baseline CT imaging of the chest, abdomen and pelvis was carried out prior to the commencement of therapy and after every 2 cycles (8 weeks). Carboplatin dose was calculated by EDTA clearance [18]. Blood samples for full blood count (FBC), biochemistry, liver function tests (LFTs) and serum CA125 test were taken prior to the commencement of therapy and before each treatment cycle. Patients were reviewed weekly during treatment for safety assessment. All safety evaluations were graded according to the National Cancer Institute Common Toxicity Criteria version 2.0. Consent was obtained from patients prior to the commencement of treatment. Ethics for the retrospective case review was obtained prior to data collection from the Northwick Park Hospital ethical review board.

## Results

### Patient 1

**Figure 1:**
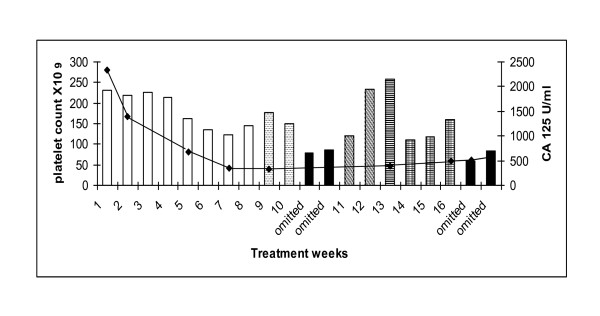
**Patient 1: platelet count (bar graph) and corresponding serum CA 125 (U/ml)**. Carboplatin AUC 3/paclitaxel 75 mg/m^2 ^(white columns). Single agent carboplatin AUC 3 (hatched columns). Dose omission (black columns). Carboplatin AUC 2/paclitaxel 20 mg/m^2 ^(diagonal lined column). Carboplatin AUC 3/paclitaxel 20 mg/m^2 ^(striped column). Carboplatin AUC 4/paclitaxel 20 mg/m^2 ^(hatched column). Serum CA 125 (solid line).

Patient 1: 63 year old diagnosed with stage 3C, grade 3 papillary serous ovarian carcinoma following suboptimal debulking surgery. She received 6 cycles of adjuvant carboplatin AUC 6 and paclitaxel 175 mg/m^2 ^(6/2002-11/2002). She relapsed eight months later (7/2003) and was treated with 6 cycles of cisplatin 80 mg/m^2 ^and etoposide 120 mg/m^2 ^(7/2003-1/2004) achieving a partial response. In 2004, she had a second relapse and received 6 cycles of single agent carboplatin AUC 6 to partial response (1/2005-6/2005). She was commenced on maintenance tamoxifen 20 mg daily from 7/2005-1/2007 until relapse when she was treated with 6 cycles of single agent carboplatin (AUC 6) (1/2007-6/2007) to partial response. Palliative radiotherapy was then administered to a rectal mass (7/2007). After a PFI of 5 months she developed disease relapse and commenced dose dense chemotherapy, carboplatin AUC 3 and paclitaxel 75 mg/m^2 ^d 1, 8 and 15 q28. Despite having a partial response on CT imaging, paclitaxel was discontinued due to the development of grade 2 peripheral neuropathy She received 2 weeks of single agent carboplatin (AUC2) but developed dose limiting thrombocytopenia (79 × 10^9^/L) resulting in two weeks dose delay. Low dose paclitaxel, 20 mg/m^2^, was then introduced as a platelet-sparing agent. The carboplatin dose was subsequently increased to AUC 3 (week 7) and then AUC 4 (week 8 and 9). She developed grade 2 thrombocytopenia requiring a 2 week delay. Carboplatin dose was reduced to AUC 3 and the patient completed 18 cycles of dose dense therapy. Serum Ca125 levels decreased from 2332 to 564 U/mL during the course of treatment and her CT confirmed partial response with a significant decrease in the size and number of both subcapsular and intrahepatic metastases (Figure 1). The patient did not experience further thrombocytopenia and her neuropathic symptoms gradually improved whilst on treatment.

### Patient 2

**Figure 2:**
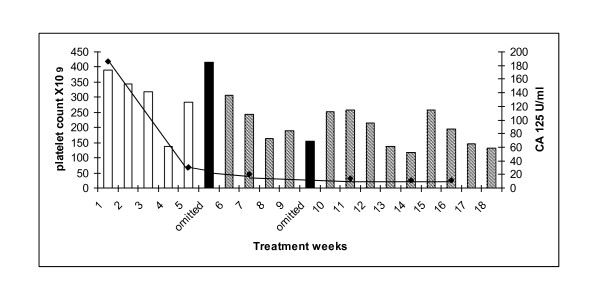
**Platelet count and corresponding corresponding serum CA 125 patient 2**. Carboplatin AUC 3/paclitaxel 75 mg/m^2 ^(white bars). Dose omission (black columns). Carboplatin AUC 3/paclitaxel 20 mg/m^2 ^(hatched bars). Serum CA 125 (solid line).

62 year old woman with stage 3C, grade 3 serous ovarian adenocarcinoma diagnosed following optimal debulking surgery in 2007 The patient received 6 cycles of adjuvant chemotherapy with carboplatin AUC 6 and paclitaxel 175 mg/m^2 ^(4/2007-8/2007). Following a PFI of five months she developed progressive disease, with bilateral hydronephrosis requiring ureteric stents. She was commenced on weekly carboplatin AUC 3 and paclitaxel 75 mg/m^2^, however her pre-existing grade 2 neuropathy significantly worsened after 5 weekly cycles, and the paclitaxel dose was reduced to 20 mg/m^2^. She completed 18 cycles of therapy with no episodes of thrombocytopenia and without any further deterioration in her neuropathic symptoms. At the end of treatment she had had a partial response both by CT and serum CA125 criteria, 186 to 12 U/mL (Figure 2).

### Patient 3

**Figure 3:**
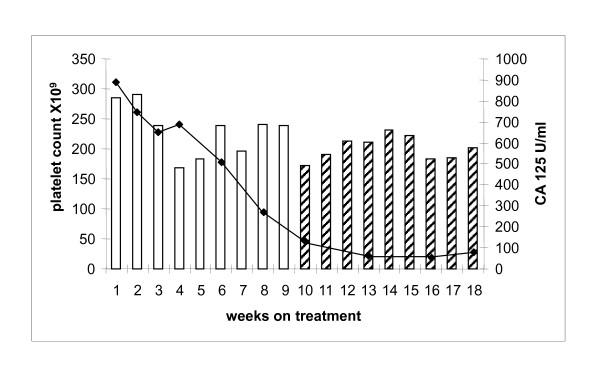
**Platelet count and corresponding corresponding serum CA 125 patient 3**. Carboplatin AUC 3/paclitaxel 75 mg/m^2 ^(white bars). Carboplatin AUC 3/paclitaxel 20 mg/m^2 ^(hatched bars). Serum CA 125 (solid line).

65 year old woman with stage 3C grade 3 serous ovarian cancer in 2007. Four cycles of neoadjuvant carboplatin AUC 6 and paclitaxel 175 mg/m^2 ^were administered followed by delayed primary surgery. She then received a further 3 cycles of carboplatin AUC 6 and paclitaxel 175 mg/m^2 ^(2/2007-9/2007). Partial response was noted on the end of treatment CT scan. Serum Ca125 started increasing 4 months following treatment completion with progressive disease confirmed on CT (1/2008). She was then commenced on weekly carboplatin AUC 3 and paclitaxel 75 mg/m^2 ^4 months after last platinum therapy. She tolerated the first 9 cycles well, however, she subsequently developed clinically significant grade 2 peripheral neuropathy and the dose of paclitaxel was reduced to 20 mg/m^2^. The patient had no delays for haematological toxicity and her peripheral neuropathy resolved on the reduced dose of paclitaxel (Figure 3). She completed 18 cycles of treatment following which CT scan confirmed PR and serum Ca125 decreased from 891 to 78 U/mL.

### Patient 4

**Figure 4:**
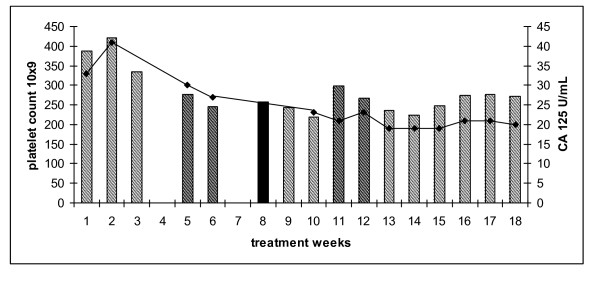
**Platelet count of patient 4 treated with weekly carboplatin AUC 3 and paclitaxel 20 mg/m^2 ^(hatched bars)**. Single agent carboplatin AUC 3 (solid bar). Serum CA 125 (U/ml)(solid line).

52 year old diagnosed in 2008 following suboptimal debulking surgery for grade 3, stage, 3C serous ovarian cancer. She developed platinum refractory disease on restaging CT scan following 3 cycles of carboplatin AUC 6 and paclitaxel 175 mg/m^2 ^(6/2008-8/2008). She also complained of grade 2 peripheral neuropathy. The patient commenced carboplatin AUC 3 and paclitaxel 20 mg/m^2 ^weekly. Paclitaxel was omitted for one week (week 8) for grade 3 arthralgia (solid colour, Figure 4) and reintroduced in subsequent cycles. She encountered no delays or dose reductions for toxicity. Furthermore, her neuropathic symptoms resolved during the course of her weekly treatment. Whilst an interim assessment suggested anti-tumour activity, at the end of 18 cycles the patient had progressive disease on CT examination.

### Patient 5

**Figure 5:**
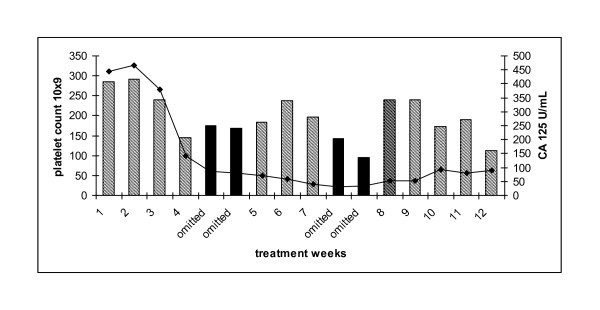
**Platelet count for patient 5 treated with weekly carboplatin AUC 3 and paclitaxel 20 mg/m^2 ^(hatched bars) and corresponding serum CA 125 (U/ml)(solid line)**. Dose omission (black bars).

76 year old diagnosed in 2006 with a grade 2, stage 3 serous adenocarcinoma of primary peritoneal origin following an omental biopsy. She was deemed to be of high operative risk because of multiple co-morbidities and received four cycles of neoadjuvant carboplatin AUC 5 followed by delayed primary surgery and 3 further cycles of carboplatin (9/2006-4/2007). She developed progressive disease (10/2007) and commenced 3-weekly paclitaxel 175 mg/m^2 ^to stable disease (10/2007-2/2008). Treatment was complicated by grade 3 peripheral neuropathy. Three months following completion of therapy she developed progressive disease and commenced on carboplatin AUC 5 in combination with liposomal doxorubicin 40 mg/m^2 ^completing 6 cycles (5/2008-11/2008) with a partial response seen on CT. She experienced disease relapse 6 months later and was commenced on weekly carboplatin AUC 3 and paclitaxel 20 mg/m^2^. Therapy was delayed for 2 weeks because of neutropaenia (week 3 and 4, although there was no thrombocytopenia) and she was commenced on G-CSF 300 μg days 2-5 with each subsequent cycle. Treatment was also delayed for 2 weeks for a tooth extraction (weeks 8 and 9). The last two weeks of treatment were omitted as she developed varicella zoster infection. Her platelet count and serum CA 125 are shown (Figure 5). Her neuropathic symptoms did not worsen during the course of treatment. At the end of 16 cycles the patient had stable disease on CT examination and serum CA 125 reduced from 465 to 89 U/ml.

### Patient 6

**Figure 6:**
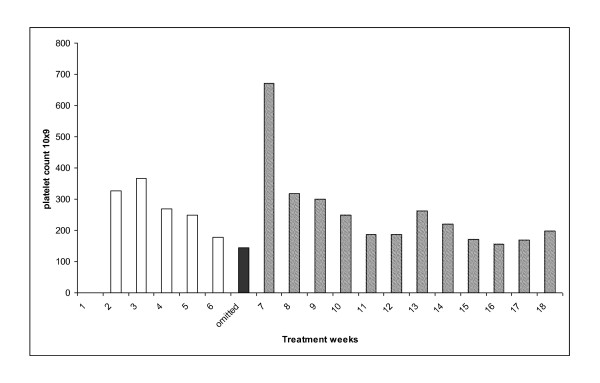
**Platelet count and corresponding corresponding serum CA 125 patient 6**. Carboplatin AUC 3/paclitaxel 75 mg/m^2 ^(white bars). Carboplatin AUC 3/paclitaxel 20 mg/m^2 ^(hatched bars). Dose omission (black bars). Serum CA 125.

57 year old woman with a stage 1A, grade 1 granulosa cell tumour of the ovary diagnosed following optimal debulking surgery in 2000. She represented three years later with recurrent pelvic and peritoneal disease. She received three cycles of bleomycin, etoposide and cisplatin (3/2004-5/2004) however interval CT scan showed no response to therapy, and she underwent complete macroscopic debulking. In August 2005 she underwent further debulking surgery for hepatic and splenic metastases. In September 2008 she developed peritoneal and hepatic metastases. She commenced 3 weekly carboplatin AUC 6 and paclitaxel 175 mg/m^2 ^(3/2009-5/2009). Interval CT scan after 3 cycles of therapy confirmed progressive disease and she commenced weekly carboplatin AUC3 and paclitaxel 75 mg/m^2^. Her pre-existing neuropathy significantly worsened (grade 2) after 7 weekly cycles, and the paclitaxel dose was reduced to 20 mg/m^2^. She completed 18 cycles of therapy with no episodes of thrombocytopaenia and without any further deterioration in her neuropathic symptoms (Figure 6). At the end of treatment she had had a partial response by CT criteria.

### Patient 7

**Figure 7:**
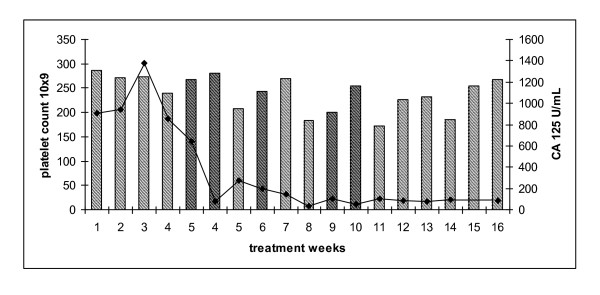
**Platelet count and corresponding corresponding serum CA 125 patient 7**. Carboplatin AUC 3/paclitaxel 20 mg/m^2 ^(hatched bars). Serum CA 125 (solid line).

Patient 7 was a 57 year old woman diagnosed with a stage 3C serous ovarian cancer following optimal debulking surgery in 2006. She received 6 cycles of adjuvant chemotherapy, carboplatin AUC 6 and paclitaxel 175 mg/m^2 ^(10/2006-2/2007) A year later she developed progressive disease and received six cycles of carboplatin AUC 6 and gemcitabine 1000 mg/m^2 ^(1/2008-6/2008). In October 2008 she developed progressive disease and completed six cycles of liposomal doxorubicin in February 2009 (10/2008-3/2009). After a PFI of 3 months, she developed progressive disease and commenced treatment with weekly carboplatin AUC 3 and paclitaxel 75 mg/m^2 ^June 2009. However her pre-existing neuropathy significantly worsened (grade 2) after 5 weekly cycles, and the paclitaxel dose was reduced to 20 mg/m^2 ^(Figure 7). She completed 16 cycles of therapy with no episodes of thrombocytopenia and without any further deterioration in her neuropathic symptoms. At the end of treatment she had had a partial response on CT and serum CA 125 reduced from 911 to 83 U/ml.

## Discussion

Several chemotherapeutic agents such as topotecan, gemcitabine, liposomal doxorubicin, paclitaxel and etoposide have been used in the treatment of platinum-resistant disease with unexciting response rates in the range of 6-15% [19-21]. Data previously published by our group and others suggest that the use of use of extended dose-dense chemotherapy results in response rates of 40-60% in this otherwise poor prognosis group [22-24]. Extended dose-dense therapy affords the opportunity to effectively and tolerably treat conventionally platinum-resistant patients with platinum again. This is of importance as platinum resistance ultimately becomes the dominant problem for most patients with ovarian cancer. It is well established that effective administration of single agent carboplatin may be compromised by dose-limiting thrombocytopenia, which may be circumvented by the platelet sparing effect of paclitaxel. However, a limitation in retreating patients with dose dense combination therapy is the development of peripheral neuropathy either because of previous taxane therapy or other co-morbidities such as diabetes mellitus. In this case series we have shown that the addition of low dose paclitaxel exerts a platelet sparing effect and does not worsen the clinical symptoms of peripheral neuropathy, and did not impact negatively on the dose intensity or the tumour response to carboplatin. The major limitation of this report is the small samples size with only seven patients included in this hypothesis generating study. However the results suggest that this approach requires further investigation in the form of a formal randomised phase II study, and would be strengthened by the inclusion of planned formal nerve conduction studies in .such a study.

The exact mechanism by which paclitaxel exerts its platelet sparing effect remains unclear. Pertusini and colleagues reported that P-glycoprotein-mediated efflux of paclitaxel, perhaps in association with glutathione S-transferase-mediated detoxification of carboplatin, results in the relative sparing of marrow colony-forming units-megakaryocytes after exposure to carboplatin and paclitaxel. Furthermore, the authors report high levels of circulating serum thrombopoietin in patients receiving combination therapy which was felt to be derived from the marrow stroma. The authors hypothesise therefore that there is relative sparing of megakaryocytes due to reduced drug exposure and these cells are then in turn stimulated by stromal derived thrombopoietin [25]. As the mechanism for this protection is not well understood, there are theoretical concerns that this platelet sparing effect could also protect the tumour. However, several studies including the combined analysis of the ICON4/AGO OVAR 2.2 study showed no survival disadvantage for the combination arm thereby providing some reassurance on this issue [26].

In the present case series we demonstrate the clinically platelet protective effect of low dose paclitaxel in patients receiving dose dense weekly carboplatin. In all patients the dose of paclitaxel was reduced because of peripheral neuropathy which did not worsen during low dose weekly paclitaxel therapy. We did not feel it was ethical to commence with a low dose of paclitaxel in patients not previously exposed to this drug because of the theoretical problem of developing resistance to paclitaxel. In patient 1, paclitaxel was initially omitted because of neuropathy and an expected abrupt reduction in platelets was noted despite a concurrent reduction in carboplatin dose to AUC2. Low dose paclitaxel was then introduced. In order to ascertain the maximal dose of weekly carboplatin that could be administered with low dose paclitaxel, the dose of carboplatin was slowly increased to an AUC 4 when the patient then developed thrombocytopaenia suggesting that AUC 3 was the likely maximal tolerated dose of carboplatin and this dose was subsequently used in the other six patients successfully. As indicated in the figures, the majority of patients received at least 8 weeks of weekly therapy with maintenance of platelet counts suggesting the utility of this regimen in this population group.

Based on these results, we suggest that with the approach of maximal carboplatin dose and low dose paclitaxel, it may be possible to create an active dose-dense platinum-focussed "scaffold" that can be used to feasibly integrate novel agents that target the reversal of platinum resistance [27]. With the realisation that delivering carboplatin AUC 3 weekly for 18 cycles is achievable and active in platinum resistant disease, a feasibility study is planned to identify the minimum dose of paclitaxel that is platelet-sparing. This strategy seeks to maximise delivered weekly platinum whilst minimising paclitaxel related toxicities, thereby maximising "space" within the scaffold" to absorb potential toxicities of targeted molecular therapies that reverse platinum resistance singly or in combination, since these therapies will bring their own toxicities to the regimen.

Investigators have recently begun to suggest that the response in weekly carboplatin and paclitaxel therapy is principally due to weekly paclitaxel, as judged by the weekly paclitaxel data [28]. This case series demonstrates that firstly, dose dense carboplatin is feasible in carboplatin focussed scaffolds, and secondly, that from the response profile of these patients, carboplatin given in this way is highly active even with very low dose paclitaxel. A potential therapeutic space is to sequence this approach after patients relapse from weekly paclitaxel; there is a rich seam of targeted therapeutics that may be highly effective in reversing acquired platinum resistance in ovarian cancer in this context that require urgent testing utilising this approach.

## Conclusion

In the present case series we have demonstrated that the addition of low dose paclitaxel allows delivery of weekly dose dense carboplatin AUC 3 by maintaining platelet counts without compromising dose dense carboplatin mediated tumour response. We have shown this regimen to be well tolerated and effective, in this otherwise poor prognostic group. Given the disappointing responses to non platinum agents in this disease setting we feel that this regimen merits further investigation as part of a larger study. We conclude that this regimen is feasible and active, and could be formally developed as a "platinum-focussed dose-dense scaffold" into which targeted therapies that reverse platinum resistance can be incorporated.

## Competing interests

The authors declare that they have no competing interests.

## Authors' contributions

RS and JG equally contributed to the study design, data collection and analysis and preparation and approval of final manuscript. SB contributed to the acquisition of data and preparation of the manuscript. HG contributed to the study design and preparation of the manuscript. All authors read and approved the final manuscript

## Pre-publication history

The pre-publication history for this paper can be accessed here:

http://www.biomedcentral.com/1471-2407/11/289/prepub

## References

[B1] Piver MS Baker TR Piedmonte M Sandecki AM Epidemiology and etiology of ovarian cancer Semin Oncol 1991 18 3 177 185 2042059

[B2] Neijt JP ten Bokkel Huinink WW van der Burg ME van Oosterom AT Willemse PH Vermorken JB van Lindert AC Heintz AP Aartsen E van Lent M Long-term survival in ovarian cancer. Mature data from The Netherlands Joint Study Group for Ovarian Cancer Eur J Cancer 1991 27 11 1367 1372 10.1016/0277-5379(91)90011-2 1835850

[B3] Angioli R Palaia I Damiani P Montera R Benedetti Panici P [Up-date on cytoreductive surgery in the management of advanced ovarian cancer] Minerva Ginecol 2006 58 6 459 470 17108876

[B4] Markman M Rothman R Hakes T Reichman B Hoskins W Rubin S Jones W Almadrones L Lewis JL Jr Second-line platinum therapy in patients with ovarian cancer previously treated with cisplatin J Clin Oncol 1991 9 3 389 393 1999708 10.1200/JCO.1991.9.3.389

[B5] van der Burg ME Hoff AM van Lent M Rodenburg CJ van Putten WL Stoter G Carboplatin and cyclophosphamide salvage therapy for ovarian cancer patients relapsing after cisplatin combination chemotherapy Eur J Cancer 1991 27 3 248 250 10.1016/0277-5379(91)90507-A 1827305

[B6] Eisenhauer EA ten Bokkel Huinink WW Swenerton KD Gianni L Myles J van der Burg ME Kerr I Vermorken JB Buser K Colombo N European-Canadian randomized trial of paclitaxel in relapsed ovarian cancer: high-dose versus low-dose and long versus short infusion J Clin Oncol 1994 12 12 2654 2666 7989941 10.1200/JCO.1994.12.12.2654

[B7] van der Burg ME van der Gaast A Vergote I Burger CW van Doorn HC de Wit R Stoter G Verweij J What is the role of dose-dense therapy? Int J Gynecol Cancer 2005 15 Suppl 3 233 240 16343238 10.1111/j.1525-1438.2005.00432.x

[B8] Vasey PA "Dose dense" chemotherapy in ovarian cancer Int J Gynecol Cancer 2005 15 Suppl 3 226 232 16343237 10.1111/j.1525-1438.2005.00438.x

[B9] Simon R Norton L The Norton-Simon hypothesis: designing more effective and less toxic chemotherapeutic regimens Nat Clin Pract Oncol 2006 3 8 406 407 16894366 10.1038/ncponc0560

[B10] Citron ML Berry DA Cirrincione C Hudis C Winer EP Gradishar WJ Davidson NE Martino S Livingston R Ingle JN Randomized trial of dose-dense versus conventionally scheduled and sequential versus concurrent combination chemotherapy as postoperative adjuvant treatment of node-positive primary breast cancer: first report of Intergroup Trial C9741/Cancer and Leukemia Group B Trial 9741 J Clin Oncol 2003 21 8 1431 1439 10.1200/JCO.2003.09.081 12668651

[B11] Katsumata N Yasuda M Takahashi F Isonishi S Jobo T Aoki D Tsuda H Sugiyama T Kodama S Kimura E Dose-dense paclitaxel once a week in combination with carboplatin every 3 weeks for advanced ovarian cancer: a phase 3, open-label, randomised controlled trial Lancet 2009 374 9698 1331 1338 10.1016/S0140-6736(09)61157-0 19767092

[B12] Fujiwara K Aotani E Hamano T Nagao S Yoshikawa H Sugiyama T Kigawa J Aoki D Katsumata N Takeuchi M A randomized Phase II/III trial of 3 weekly intraperitoneal versus intravenous carboplatin in combination with intravenous weekly dose-dense paclitaxel for newly diagnosed ovarian, fallopian tube and primary peritoneal cancer Jpn J Clin Oncol 41 2 278 282 10.1093/jjco/hyq182 20937602

[B13] Daga H Isobe T Miyazaki M Fujitaka K Kondo K Kohno N Investigating the relationship between serum thrombopoietin kinetics and the platelet-sparing effect: A clinical pharmacological evaluation of combined paclitaxel and carboplatin in patients with non-small cell lung cancer Oncol Rep 2004 11 6 1225 1231 15138560

[B14] van Warmerdam LJ Huizing MT Giaccone G Postmus PE ten Bokkel Huinink WW van Zandwijk N Koolen MG Helmerhorst TJ van der Vijgh WJ Veenhof CH Clinical pharmacology of carboplatin administered in combination with paclitaxel Semin Oncol 1997 24 1 Suppl 2 S2-97-S92-104 9045347

[B15] Ishikawa H Fujiwara K Suzuki S Tanaka Y Kohno I Platelet-sparing effect of paclitaxel in heavily pretreated ovarian cancer patients Int J Clin Oncol 2002 7 5 330 333 12402070 10.1007/s101470200050

[B16] Akerley W Rathore R Ready N Leone L Sikov W Safran H Kennedy T A phase I study of a weekly schedule of paclitaxel and carboplatin in patients with advanced carcinoma Cancer 2002 95 9 2000 2005 10.1002/cncr.10902 12404295

[B17] Sehouli J Stengel D Elling D Ortmann O Blohmer J Riess H Lichtenegger W First-line chemotherapy with weekly paclitaxel and carboplatin for advanced ovarian cancer: a phase I study Gynecol Oncol 2002 85 2 321 326 10.1006/gyno.2002.6623 11972395

[B18] Calvert AH Newell DR Gumbrell LA O'Reilly S Burnell M Boxall FE Siddik ZH Judson IR Gore ME Wiltshaw E Carboplatin dosage: prospective evaluation of a simple formula based on renal function J Clin Oncol 1989 7 11 1748 1756 2681557 10.1200/JCO.1989.7.11.1748

[B19] Swisher EM Mutch DG Rader JS Elbendary A Herzog TJ Topotecan in platinum- and paclitaxel-resistant ovarian cancer Gynecol Oncol 1997 66 3 480 486 10.1006/gyno.1997.4787 9299264

[B20] Mutch DG Surgical management of ovarian cancer Semin Oncol 2002 29 1 Suppl 1 3 8 11840413 10.1053/sonc.2002.31589

[B21] Gordon AN Fleagle JT Guthrie D Parkin DE Gore ME Lacave AJ Recurrent epithelial ovarian carcinoma: a randomized phase III study of pegylated liposomal doxorubicin versus topotecan J Clin Oncol 2001 19 14 3312 3322 11454878 10.1200/JCO.2001.19.14.3312

[B22] de Jongh FE de Wit R Verweij J Sparreboom A van den Bent MJ Stoter G van der Burg ME Dose-dense cisplatin/paclitaxel. a well-tolerated and highly effective chemotherapeutic regimen in patients with advanced ovarian cancer Eur J Cancer 2002 38 15 2005 2013 10.1016/S0959-8049(02)00242-3 12376205

[B23] Van der Burg ME Phase II study of weekly paclitaxel carboplatin in the treatment of progressive ovarian cancer Journal of Clinical Oncology 2004 22 14S 5058

[B24] Sharma R Graham J Mitchell H Brooks A Blagden S Gabra H Extended weekly dose-dense paclitaxel/carboplatin is feasible and active in heavily pre-treated platinum-resistant recurrent ovarian cancer Br J Cancer 2009 100 5 707 712 10.1038/sj.bjc.6604914 19223898 PMC2653750

[B25] Pertusini E Ratajczak J Majka M Vaughn D Ratajczak MZ Gewirtz AM Investigating the platelet-sparing mechanism of paclitaxel/carboplatin combination chemotherapy Blood 2001 97 3 638 644 10.1182/blood.V97.3.638 11157479

[B26] Parmar MK Ledermann JA Colombo N du Bois A Delaloye JF Kristensen GB Wheeler S Swart AM Qian W Torri V Paclitaxel plus platinum-based chemotherapy versus conventional platinum-based chemotherapy in women with relapsed ovarian cancer: the ICON4/AGO-OVAR-2.2 trial Lancet 2003 361 9375 2099 2106 12826431 10.1016/s0140-6736(03)13718-x

[B27] Stronach EA Rama N Gabra H Identification of functional modulators of platinum resistance using isogenically matched ovarian cancer cell line models 2008 AACR Annual Meeting: 2008; San Diego: Proc of the 99th Annual Meeting of the American Association for Cancer Research 200819892666

[B28] Linch M Stavridi F Hook J Barbachano Y Gore M Kaye SB Experience in a UK cancer centre of weekly paclitaxel in the treatment of relapsed ovarian and primary peritoneal cancer Gynecol Oncol 2008 109 1 27 32 10.1016/j.ygyno.2008.01.007 18262259

